# Apoptosis induction in renal cell carcinoma by TRAIL and *γ*-radiation is impaired by deficient caspase-9 cleavage

**DOI:** 10.1038/sj.bjc.6600984

**Published:** 2003-05-27

**Authors:** U Ramp, E Caliskan, C Mahotka, A Krieg, S Heikaus, H E Gabbert, C D Gerharz

**Affiliations:** 1 Institute of Pathology, Heinrich Heine University, Moorenstr. 5, D-40225 Duesseldorf, Germany

**Keywords:** renal cell carcinoma, apoptosis induction, TRAIL, ionising radiation, signal transduction pathways, caspases

## Abstract

TNF-related apoptosis-inducing ligand (TRAIL APO-2L) is a member of the TNF family and induces apoptosis in cancer cells without affecting most non-neoplastic cells. The present investigation is focused on apoptosis induction by combined exposure to TRAIL and ionising radiation (IR) in human renal cell carcinoma (RCC) cell lines. Here, we demonstrate that all RCC cell lines coexpress TRAIL and the death-inducing receptors, TRAIL-R1 and TRAIL-R2. Exposure to TRAIL alone induced marked apoptosis in three out of eight RCC cell lines. Combined exposure to TRAIL and IR resulted in a sensitisation to TRAIL-induced apoptosis in one RCC cell line only. Enhanced apoptosis induction by TRAIL in combination with IR was paralleled by an increase in PARP cleavage and activation of executioner caspase-3, whereas caspases-6 and -7 were not involved. Moreover, exposure to TRAIL and/or IR resulted in a marked activation of initiator caspase-8, possibly augmented by the observed reduction of inhibitory c-FLIP expression. In contrast to other tumour types, activation of initiator caspase-9 was not detectable in our RCC model system after exposure to TRAIL and/or IR. This lack of caspase-9 activation might be related to an impaired ‘crosstalk’ with the caspase-8 pathway as suggested by the missing Bid cleavage and to the appearance of an XIAP cleavage product known to inhibit caspase-9 activation. Deficient activation of caspase-9, therefore, might contribute to the clinically known resistance of human RCC against IR and also argues against an effective combination therapy with TRAIL and IR in this tumour type.

Resistance of malignant tumours including human renal cell carcinoma (RCC) to chemotherapy and ionising radiation (IR) remains a major problem in oncology and is, at least in part, explained by defects in apoptotic pathways (Lowe *et al*, 1993; Mulders *et al*, 1997; Müller *et al*, 1998; Zhivotovsky *et al*, 1999; Verheij and Bartelink, 2000; Igney and Krammer, 2002). Especially the pathways of IR-induced apoptosis are not well understood so far. It is thought, however, that IR-induced apoptosis is primarily executed via release of cytochrome *c* from mitochondria and subsequent activation of the caspase-9 pathway (Zhivotovsky *et al*, 1999; Belka *et al*, 2000,^2001^; Newton and Strasser, 2000; Joseph *et al*, 2001; Rudner *et al*, 2001). In addition, activation of caspase-8 has been observed during IR-induced apoptosis, which could be related to the activation of TRAIL-R2 death receptors (Belka *et al*, 2000). The corresponding TRAIL ligand (APO-2L) is a type II transmembrane protein, which induces apoptosis through the specific interaction with the two death receptors TRAIL-R1 (DR4) and -R2 (DR5), both exhibiting a death domain in their cytoplasmatic regions (for a review, see Walczak and Krammer, 2000). In addition, TRAIL also binds to the decoy receptors TRAIL-R3 (DcR1) and -R4 (DcR2), which lack a functional cytoplasmatic death domain and therefore cannot induce apoptosis. TRAIL-induced apoptosis was reported to be primarily mediated via activation of caspase-8 (Gong and Almasan, 2000; Yamanaka *et al*, 2000; Belka *et al*, 2001; Nimmanapalli *et al*, 2001; Suliman *et al*, 2001; Sun *et al*, 2001) but might also result from the proteolytic activation of Bid, which mediates the release of cytochrome *c* from mitochondria, thereby additionally activating caspase-9 (Belka *et al*, 2001; Eggert *et al*, 2001; Nimmanapalli *et al*, 2001; Seol *et al*, 2001; Suliman *et al*, 2001; Sun *et al*, 2001). The simultaneous activation of both pathways, that is, the activation of caspase-8 and caspase-9, is thought to result in an further amplification of the original apoptosis signal. Both pathways subsequently converge in the activation of executioner caspase-3, which in turn cleaves a set of cellular substrates resulting in apoptosis (Belka *et al*, 2000,^2001^; Walczak and Krammer, 2000; Joseph *et al*, 2002).

TRAIL was shown to exhibit potent antitumour activity upon systemic administration *in vivo* without the deleterious side effects on normal tissues known from CD95-ligand (Ashkenazi *et al*, 1999; Walczak *et al*, 1999; Nagata, 2000). Our group (Dejosez *et al*, 2000) and others (Ashkenazi *et al*, 1999; Pawlowski *et al*, 2000; Oya *et al*, 2001) also observed responsiveness to TRAIL-mediated apoptosis in some RCC cell lines, raising the possibility that TRAIL, either alone or in combination with other therapeutic strategies, might become a cancer therapeutic for otherwise unresponsive RCCs. Thus, a synergistic enhancement of TRAIL-mediated apoptosis by different anticancer drugs could be demonstrated in RCC cell lines suggesting that TRAIL might overcome resistance of RCC cells against anticancer drug-induced apoptosis (Dejosez *et al*, 2000; Mizutani *et al*, 2002).

The combination of TRAIL with IR might become another therapeutic option in order to overcome apoptosis resistance of tumour cells and several reports could actually demonstrate an additive or synergistic effect in different tumour models (Chinnaiyan *et al*, 2000; Gong and Almasan, 2000; Belka *et al*, 2001; Di Pietro *et al*, 2001; Kim *et al*, 2001a; Ravi *et al*, 2001). As corresponding investigations are missing for RCCs so far, we analysed the effects of combined treatment with TRAIL and IR in well-characterised human RCC cell lines of the clear cell type (Gerharz *et al*, 1993,^1994^).

## MATERIALS AND METHODS

### Cells and culture

All eight cell lines used in this study were derived from typical representatives of the clear cell (clearCa-2, -4, -6, -7, -14, -20, -22 and -23) type of RCC (Gerharz *et al*, 1993,^1994^). The cell lines were maintained with Dulbeccos's modified Eagle's medium (DMEM, Gibco, Karlsruhe, FRG), supplemented with 10% foetal calf serum (FCS), penicillin and streptomycin (=standard growth medium) and cultivated at 37°C in an atmosphere with 5% CO_2_.

### DNA extraction, polymerase chain reaction (PCR) amplification and analysis of p53 mutations

Extraction of genomic DNA was performed using the QIAmp Tissue Kit (Qiagen, Hildeu, FRG) according to the manufacturer's protocol. For amplification of p53 exons 5–9, the following oligonucleotide primers were used:

exons 5 and 6: forward: 5′-TTC CTC TTC CTG CAG TAC TC-3′
       reverse: 5′-ATG TGC AAA CCA GAC CTC AG -3′
exons 7 and 8: forward: 5′-GTG TTG TCT CCT AGG TTG GC-3′
       reverse: 5′-AAG TGA ATC TGA GGC ATA AC-3′
exon 9:    forward: 5′-GTT ATG CCT CAG ATT CAC TT-3′
       reverse: 5′-TTG AGT GTT AGA CTG GAA AC 3′

Each amplification reaction was carried out in a total volume of 50 *μ*l containing 200 ng of genomic DNA, 100 pmol of each primer, 10 nM of each dNTP, 2 U *Taq* polymerase and 1 × PCR reaction buffer (Sigma, Seelze, FRG). After an initial denaturation step at 94°C for 2 min, 35 cycles of denaturation at 94°C for 30 s, annealing at 50°C for 40 s, and extension at 72°C for 1 min as well as a last delay at 72°C for 10 min were performed on a PTC-100-thermocycler (MJ-Research, USA). After amplification, 5 *μ*l of each PCR reaction was analysed by electrophoresis on a 1.5% agarose gel and visualised by ethidium-bromide staining.

The PCR products were purified from surplus oligonucleotides using Microspin S-300 columns (Pharmacia, Erlangen, FRG). The purified PCR products were prepared for automatic sequencing using the ABI-Prism BigDye Terminator Cycle Sequencing Kit (Perkin–Elmer, Rodgau, FRG) according to the manufacturer's protocol. Sequence analysis was carried out with the sense (5′) and the antisense (3′) primers using an ABI-Prism 310 sequencer (Perkin–Elmer, Rodgau, FRG). p53 mutations were verified by an independent PCR amplification of genomic DNA followed by a repeated DNA sequencing.

### RNA extraction and reverse transcription (RT)–PCR analysis of TRAIL and its receptors

Total cellular RNA was isolated from RCC cell lines using the RNeasy kit (Qiagen, Hildeu, FRG). RNA concentration was measured by photometry at 260 nm. The quality of total cellular RNA was verified by the integrity of 18S/28S ribosomal RNA in ethidium bromide-stained agarose gels.

For monitoring expression of TRAIL and its R1-, R2-, R3- and R4-receptors, RT was performed using an RT-kit (Stratagene, La Jolla, USA), 5 *μ*g of total cellular RNA, 10 pmol of gene-specific antisense primer (TRAIL-R3) or 100 pmol random primer (TRAIL, R1, R2, R4 and GAPDH) and 5 U AMV reverse transcriptase (Promega, Madison, USA) with the corresponding RT buffer in a final volume of 30 *μ*l. The reactions were incubated at 55°C for 1 h.

The PCR mixture was composed as follows: 1.5 *μ*l of the cDNA solution as a template, 25 pmol of each gene-specific primers, 12.5 nmol of dNTPs (Stratagene, La Jolla, USA) and 2.5 U *Taq* polymerase (Sigma, Seelze, FRG) with the corresponding buffer in a final volume of 50 *μ*l.

The following primer sequences were used:

TRAIL:  forward: 5′-ACA GCA GTC AGA CTC TGA CAG GAT C-3′
     reverse: 5′-ACG GAG TTG CCA CTT GAC TTG CCA G-3′
TRAIL-R1: forward: 5′-CAG AAC GTC CTG GAG CCT GTA AC-3′
     reverse: 5′-ATG TCC ATT GCC TGA TTC TTT GTG-3′
TRAIL-R2: forward: 5′-GAT TGT ACA CCC TGG AGT GAC ATC G-3′
     reverse: 5′-CCA CAG TAA AGA CTT GCA AAC AAA CAC-3′
TRAIL-R3: forward: 5′-CTG CCA GTC CTA GCT TAC TCT G-3′
     reverse: 5′-CTG CTA CAC TTC CGG CAC ATC T-3′
TRAIL-R4: forward: 5′-GAC CCC AAG ATC CTT AAG TTC G-3′
     reverse: 5′-TGT TCT ACA CGT CCG GCA CAT C-3′
GAPDH:  forward: 5′-ACG GAT TTG GTC GTA TTG GGC G-3′
     reverse: 5′-CTC CTG GAA GAT GGT GAT GG-3′

The initial denaturation step at 94°C for 4 min was followed by 35 cycles (or 27 cycles for GAPDH) of denaturation for 1 min (TRAIL and TRAIL-R1, -R2, -R3, -R4) or 30 s (GAPDH), annealing for 1 min at 55°C (TRAIL and TRAIL-R1, -R2, -R3, -R4) or 64°C (GAPDH), extension at 72°C for 1 min, and a final extension step at 72°C for 10 min. The PCR products were separated on 3% agarose gels and the identity of the amplification products was confirmed by DNA sequencing (data not shown).

### Assessment of cell number after exposure to rhs TRAIL and/or IR

Recombinant human soluble (rhs) TRAIL (Pepro Tech, Rocky Hill USA) was added to the cultures to yield a final concentration of 10 and 100 ng ml^−1^, respectively. In an other set of experiments, cultures were exposed to IR with doses of 1 or 5 Gy. Four replicate 24-well plates were exposed to standard growth medium supplemented with 10 or 100 ng ml^−1^ rhs TRAIL or exposed to IR with 1 or 5 Gy, respectively. Moreover, two replicate 24-well plates with standard growth medium were first exposed to IR (1 or 5 Gy) and afterwards exposed to standard growth medium supplemented with 10 ng ml^−1^ rhs TRAIL. 5 × 10^4^ cells were seeded in each culture plate. As controls, two replicate 24-well plates received inocula of 5 × 10^4^ cells in standard growth medium. In each experiment, cells were harvested separately after 72 h. Cells were not refed during this period. The assessment of cell number was performed in three independent experiments (*n*=3). The number of cells harvested was counted by the trypan-blue exclusion method.

### Statistical analysis

Interactions between rhs TRAIL and IR were analysed by the fractional inhibition method as follows: when expressed as the fractional inhibition cell viability, additive inhibition produced by both inhibitors (*i*) occurs when *i*_1,2_=*i*_1_+*i*_2_; synergism when *i*_1,2_>*i*_1_+*i*_2_; and antagonism when *i*_1,2_<*i*_1_+*i*_2_.

### Morphological assessment of TRAIL-induced apoptosis

1 × 10^4^ cells were seeded in each chamber of eight-chamber slides (Nunc, Wiesbaden, FRG). After 24 h, the cells were treated with 10 or 100 ng ml^−1^ rhs TRAIL and cultured for another 72 h. As a control, tumour cells were exposed to standard growth medium. Apoptosis was determined by light microscopy of haematoxylin–eosin (HE)-stained cells showing the typical morphological signs of apoptosis, that is, chromatin condensation and/or fragmentation into apoptotic bodies (Gerharz *et al*, 1999; Ramp *et al*, 2000,^2001^).

### Western blot analysis

Ten replicate 25 cm^2^ culture flasks, each containing inocula of 1 × 10^6^ cells, were exposed to standard growth medium supplemented with rhs TRAIL (10 ng ml^−1^) or exposed to IR (1 Gy), respectively. Moreover, five replicate 25 cm^2^ culture flasks with standard growth medium were first exposed to IR (1 Gy) and afterwards exposed to standard growth medium supplemented with rhs TRAIL (10 ng ml^−1^). As controls, six replicate 25 cm^2^ culture flasks received inocula of 1 × 10^6^ cells in standard growth medium. In each experiment, cells were harvested separately after 0.5, 3, 6, 12 and 24 h or after 12 h (for analysis of c-FLIP, FADD, Bid, phosphorylated Akt, XIAP and survivin). Cells were not refed during this period.

For Western blot analysis, tumour cells were lysed in a buffer containing 100 mM NaCl, 10 mM Tris-HCl (pH 7.6), 1 mM EDTA (pH 8.0), 1 *μ*g ml^−1^ aprotinin, 100 *μ*g ml^−1^ phenylmethylsulphonyl fluoride and 1% NP40. Protein (50 *μ*g) was analysed by SDS–PAGE and protein expression levels were determined by immunoblotting with the following monoclonal or polyclonal antibodies: caspase-3 (1 : 150, Cell Signaling Technology, Beverly, USA), caspase-6 (1 : 1000, Cell Signaling Technology, Beverly, USA) caspase-7 (clone B94-1; 1 : 1000, Pharmingen, San Diego, USA), caspase-8 (clone 1C12; 1 : 1000, Cell Signaling Technology, USA), caspase-9 (clone 1–2; 1 : 500, Oncogene, La Jolla, USA), Bid (1 : 500, Biosource, Camarillo, FRG), phosphorylated Akt (clone 4E2; 1 : 1000, Cell Signaling Technology, Beverly, USA), Fas-associated death domain-containing protein (FADD) (clone A66-2; 1 : 1000, Pharmingen, San Diego, USA), PARP (clone 4C10-5; 1 : 1000, Pharmingen, San Diego, USA), X-linked inhibitor of apoptosis proteins (XIAP) (clone 48; 1:250, Transduction Laboratories, Lexington, USA), Survivin (1 : 1000, Novus Biologicals, Littleton, USA) and c-FLICE-inhibitory protein (FLIP) (clone NF6, 1 : 10, gift from Professor Dr P Krammer, DKFZ, Heidelberg).

For detection, the ECL detection system (Fa. Amersham, Freiburg, FRG) was used according to the manufacturer's instructions. Equal loading of the gels was confirmed both by Commassie-blue staining of control gels and by reincubation of the filters with a monoclonal antibody for *α*-tubulin (clone B-5-1-2; 1 : 5000, Sigma-Aldrich, Seelze, FRG).

## RESULTS

### TRAIL-mediated apoptosis in human RCCs

**Figure 1:**
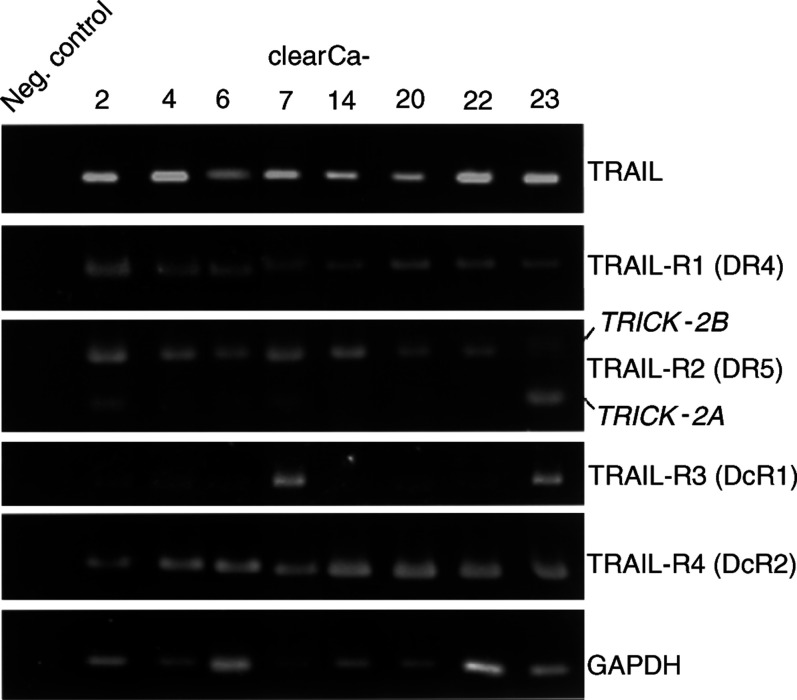
Expression of TRAIL and its receptors in eight human RCC cell lines by RT–PCR. TRICK-2A and TRICK-2B are alternative splicing variants of TRAIL-R2. GAPDH detection was used as control for integrity of RNA and RT–PCR reactions.

Reverse transcription–polymerase chain reaction RT–PCR analysis revealed coexpression of TRAIL ligand as well as apoptosis-inducing TRAIL-R1 and -R2 in all RCC cell lines (Figure 1

). Moreover, two different specific amplification products (120 and 217 bp) of TRAIL-R2 were detectable in all cell lines and verified as TRICK-2A and TRICK-2B by sequence analysis (data not shown). All RCCs expressed antagonistic TRAIL-R3 and -R4 with weak signals for TRAIL-R3 in the majority of cell lines (Figure 1).

**Figure 2:**
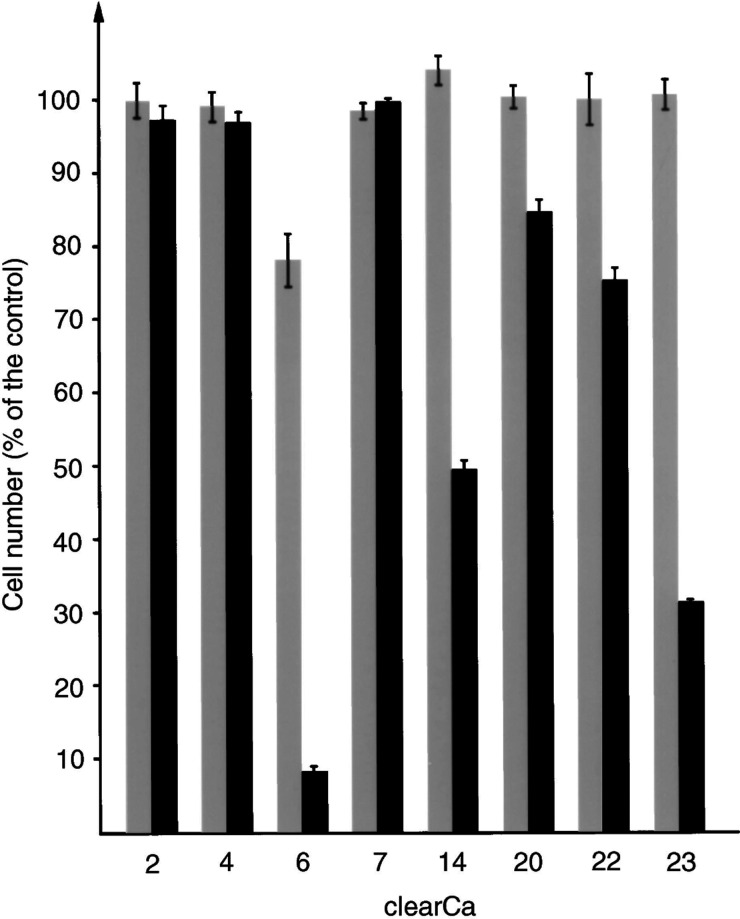
Response of human RCC cell lines to TRAIL-induced cell death. Cells were cultured for 72 h in the presence of TRAIL (grey bars:10 ng ml^−1^ TRAIL; black bars: 100 ng ml^−1^ TRAIL) and the number of surviving, nonapoptotic cells is demonstrated in percentage of viable cells in the control (bars represent the s.d).

**Figure 3:**
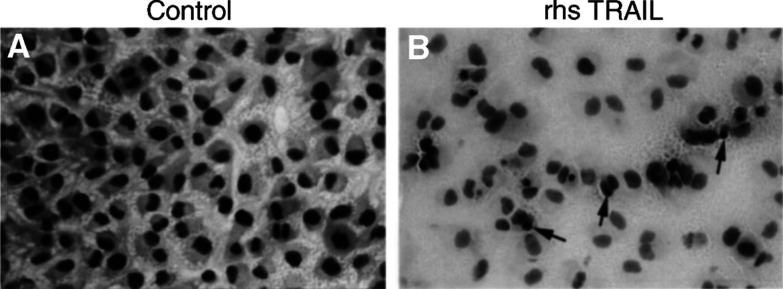
Marked induction of apoptosis (arrows) and marked reduction of cell density in clearCa-6 cells after exposure to TRAIL (100 ng ml^−1^) (**B**) when compared to the control (**A**). (Before TRAIL treatment, about 2% of the cells exhibited morphological features of apoptosis. The proportion of apoptotic cells after TRAIL treatment, however, cannot exactly be determined, because most apoptotic cells detached from the bottom of the culture flask and formed a not exactly quantifiable mass of cell detritus in the supernatant).

To evaluate the extent of TRAIL-mediated apoptosis and the functionality of the TRAIL-signalling pathway, we used rhs TRAIL, thereby confronting our RCC cell lines with an uniform apoptotic signal. After exposure to rhs TRAIL (10 or 100 ng ml^−1^) for 72 h, a heterogeneous response was observed with a marked decrease of cell viability in only three out of eight RCC cell lines (clearCa-6, -14 and –23) (Figure 2

). The decrease of cell viability after exposure to TRAIL was paralleled by a marked induction of apoptosis (Figure 3

), as became evident from light microscopical evaluation of HE-stained cells, which had been shown to be a highly sensitive and specific method for the assessment of apoptosis (Gerharz *et al*, 1999; Ramp *et al*, 2000,^2001^).

### p53 status

As wild-type p53 has been reported to be involved in apoptosis induction after exposure to TRAIL and/or IR (Lowe *et al*, 1993; Chinnaiyan *et al*, 2000), we additionally defined the mutational status of p53 in our RCC cell lines. Sequencing of p53 exons 5–9, which are the most commonly affected hot spot regions for p53 mutations in human RCC (Reiter *et al*, 1993), revealed p53 exon mutations in two of eight RCC cell lines only (Table 1

**Table 1:** p53 mutation status

**Cell line**	**p53 (wild-type (wt) or mutation (mt))**
2	mt in noncoding region: codon 306: CGA→CAA
4	mt: exon 8, codon 273: CGA→CAT
6	mt: exon 8, codon 290: CGC→CAC
7	wt
14	wt
20	wt
22	mt in noncoding region: codon 331: CTT→CTC
23	wt

). The p53 status, however, could not be correlated with the sensitivity to TRAIL-induced apoptosis. Thus, mutant p53 was observed in clearCa-6, which is highly TRAIL-sensitive, but also in clearCa-4, which is TRAIL-resistant.

### Ionising radiation sensitises to TRAIL-induced apoptosis in a minority of RCCs

**Figure4:**
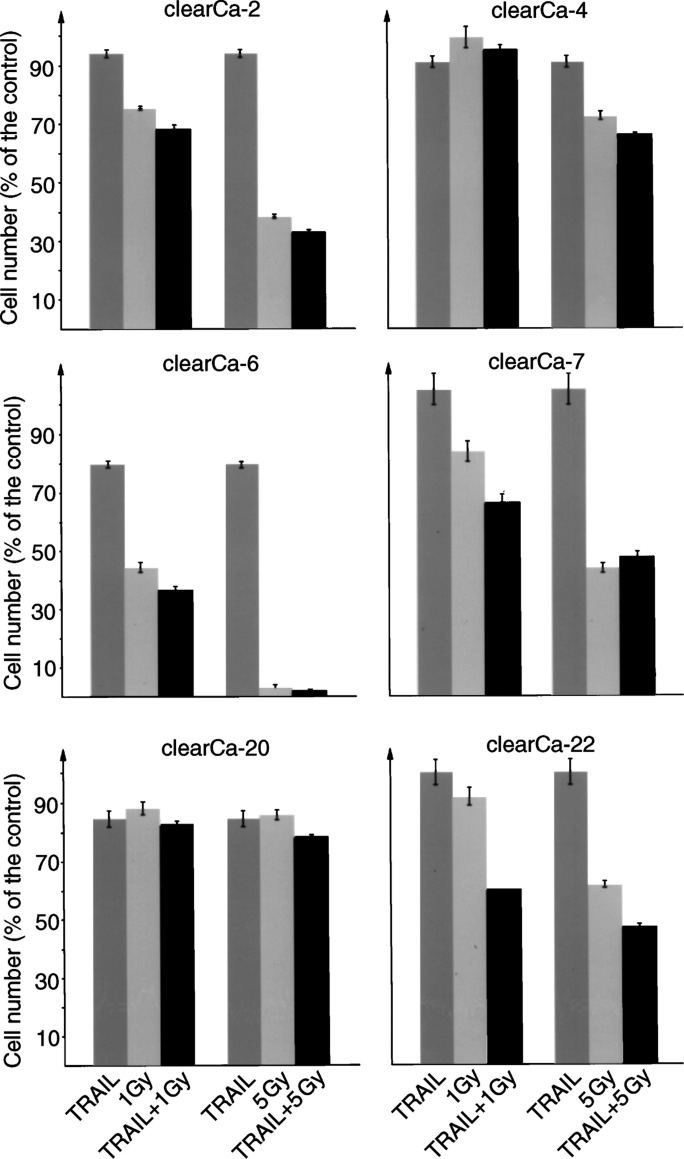
Sensitisation to TRAIL-induced apoptosis by IR in one (clearCa-22) out of six human RCC cell lines. RCC cell lines were cultured for 72 h in the presence of TRAIL (10 ng ml^−1^) or IR (1 or 5 Gy) alone or in combination and the number of surviving, nonapoptotic cells is demonstrated in percentage of viable cells in the control (bars represent the s.d).

**Figure 5:**
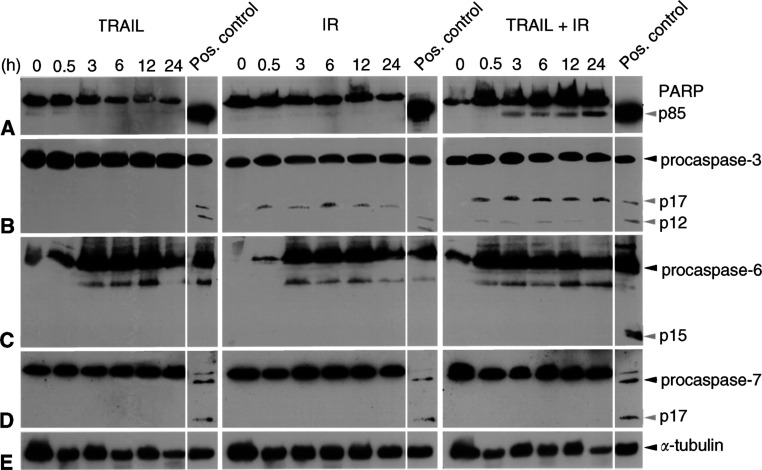
Western blot analysis demonstrating marked cleavage of PARP (p85; **A**) and procaspase-3 (p17/p12; **B**) into its active fragments after *combined* exposure of clearCa-22 cells to TRAIL (10 ng ml^−1^) and IR (1 Gy). Exposure to TRAIL or IR *alone* did not result in increased cleavage. No cleavage products of procaspases-6 (p15; **C**) and -7 (p17; **D**) in clearCa-22 cells after exposure to TRAIL or IR alone and in combination. (cleavage of PARP as well as the procaspase-3, -6, and -7 in J16 cells after exposure to CH11 (500 ng ml^−1^) was used as a positive control). Expression of *α*-tubulin (**E**) shows equal amounts of protein loaded in each lane.

To evaluate a possible increase in sensitivity against TRAIL-induced apoptosis by IR, we selected TRAIL-resistant RCC cell lines (clearCa-2, -4, -7, -20 and -22) and the most TRAIL-sensitive cell line (clearCa-6) for the combined exposure to TRAIL (10 ng ml^−1^) and clinically relevant IR doses (1 or 5 Gy). In these experiments, combined exposure to TRAIL and IR resulted in a sensitisation effect with marked enhancement of apoptosis in one RCC cell line only (clearCa-22) (Figure 4

). Interestingly, this cell line did not harbour a mutation in the p53 ‘hot spot’ exons and had shown increased expression of TRAIL-R2 protein after IR by Western blot analysis (data not shown). In accordance, a strong enhancement of apoptosis induction also became evident from a marked increase in PARP cleavage after combined exposure of clearCa-22 to TRAIL and IR, whereas exposure to either TRAIL or IR alone did not result in increased levels of PARP cleavage (Figure 5A

). Combined exposure of clearCa-22 to TRAIL and IR also resulted in a marked caspase-3 activation, whereas exposure to either TRAIL or IR alone induced no (TRAIL) or a weak (IR) caspase-3 cleavage only (Figure 5B). In contrast, activation of executioner caspase-6 and -7 was not detectable (Figure 5C, D).

### Cleavage of caspase-8, but not of caspase-9 triggers TRAIL- and/or IR-mediated apoptosis in RCC

Since the sensitisation to apoptosis induction after combined exposure to TRAIL and IR may be due to the simultaneous activation of the caspase-8 *and* -9 pathways, we next analysed the activation of these distinct apoptosis pathways in clearCa-22.

**Figure 6:**
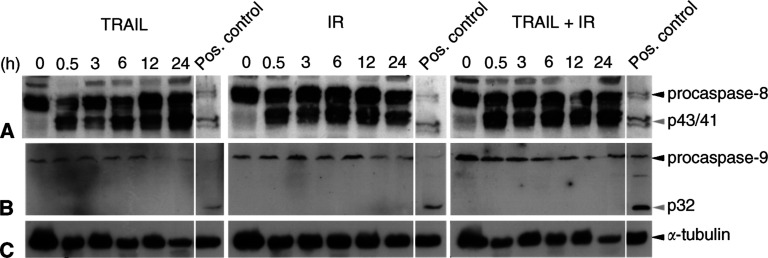
Western blot analysis demonstrating marked cleavage of procaspase-8 (**A**) into its active p43- and p41-fragments after exposure of clearCa-22 cells to TRAIL (10 ng ml^−1^) or IR (1 Gy) alone and in combination. No cleavage of procaspase-9 (**B**) in clearCa-22 cells after exposure to TRAIL or IR alone and in combination. (cleavage of procaspase-8 and -9 in J16 cells after exposure to CH11 (500 ng ml^−1^) was used as a positive control). Expression of *α*-tubulin (**C**) shows equal amounts of protein loaded in each lane.

**Figure 7:**
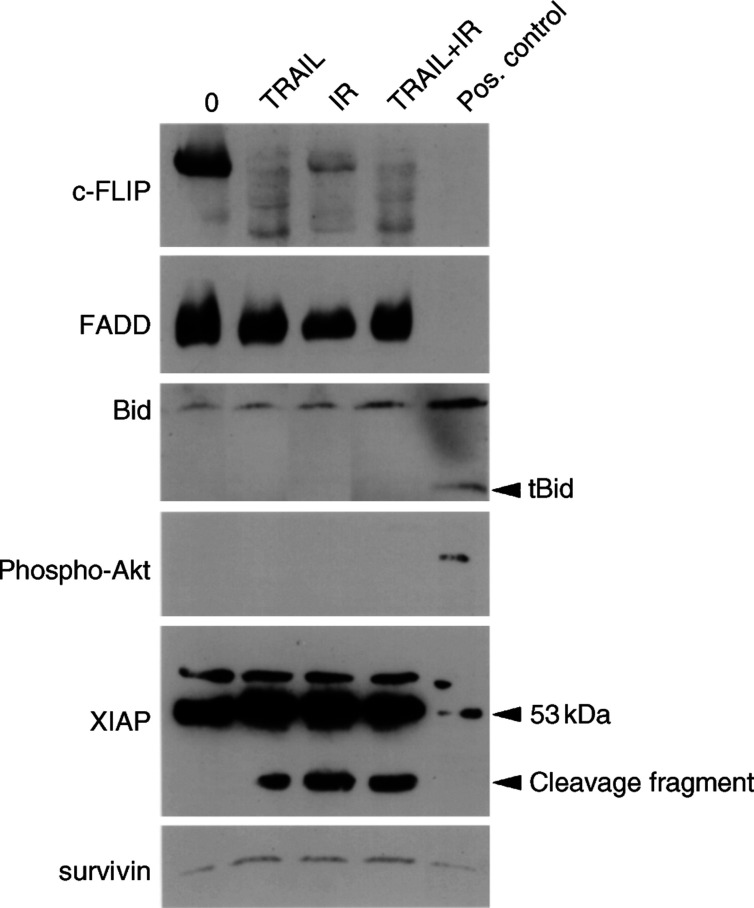
Western blot analysis of apoptosis-regulating proteins in clearCa-22 after exposure to TRAIL (10 ng ml^−1^) or IR (1 Gy) alone and in combination for 12 h. Reduced expression of c-FLIP after exposure to TRAIL and/or IR, whereas expression of FADD and survivin remained unchanged when compared with the control (0). No cleavage of Bid into tBid and absence of active phosphorylated Akt protein after exposure to TRAIL and/or IR. High levels of XIAP protein (53 kDa) before and after exposure to TRAIL and/or IR and appearance of an XIAP cleavage product after exposure to TRAIL and/or IR. (Cleavage of Bid into tBid in J16 cells after exposure to CH11 was used as a positive control; expression of phosphorylated Akt in PDGF-induced NIH-3T3 cells was used as a positive control, as recommended by the manufacturer).

As shown in Figure 6A

, cleavage of initiatior caspase-8 into its fragments (p43 and p41) became evident in clearCa-22 after exposure to TRAIL and/or IR. Of note, caspase-8 activation after combined exposure to TRAIL and IR did not exceed the effects of each treatment alone, although only combined exposure had resulted in an increased cleavage of caspase-3. Interestingly in this context, expression of caspase-8-inhibitory c-FLIP (Irmler *et al*, 1997; Wang *et al*, 2000) was markedly reduced after exposure to TRAIL or IR alone and in combination, thereby probably contributing to the activation of caspase-8 (Figure 7

). In contrast, no unequivocal differences in the expression of FADD became evident after exposure to TRAIL and/or IR (Figure 7).

In contrast to activation of the caspase-8 pathway, activation of the caspase-9 pathway was not detectable in clearCa-22 at all (Figure 6B). Since the caspase-9 pathway was shown to be functionally involved in IR-induced apoptosis in other tumour models (Newton and Strasser, 2000; Joseph *et al*, 2001; Rudner *et al*, 2001), the impaired activation of caspase-9 might contribute to the well-known resistance of RCC to IR-induced apoptosis.

Moreover, our investigations provided first clues to the mechanisms of deficient caspase-9 activation in RCC. Thus, cleavage of Bid into tBid, which is the connecting link between the caspase-8 and caspase-9 pathways of apoptosis induction (Li *et al*, 1998), could not be observed after exposure to TRAIL and/or IR (Figure 7). Failure of Bid cleavage could not be attributed to the presence of active phosphorylated Akt protein, which was not detectable at all (Figure 7). Moreover, high levels of XIAP expression were observed before and after exposure to TRAIL and/or IR (Figure 7) and, more importantly, a cleavage product of XIAP, which is known to inhibit caspase-9 activation (Deveraux *et al*, 1999), appeared after exposure to TRAIL and/or IR (Figure 7). In contrast, only low expression levels of antiapoptotic survivin, known to inhibit activation of caspase-3, -7 and -9, were found before and after exposure to TRAIL and/or IR (Figure 7).

## DISCUSSION

Ionising radiation is thought to exert its therapeutic effects, at least in part, by induction of apoptosis in tumour cells (Brown and Wouters, 1999; Zhivotovsky *et al*, 1999; Newton and Strasser, 2000; Verheij and Bartelink, 2000). As TRAIL has recently been shown to induce apoptosis effectively in tumour cells (Ashkenazi *et al*, 1999; Walczak *et al*, 1999; Dejosez *et al*, 2000; Nagane *et al*, 2000; Walczak and Krammer, 2000) but not in normal cells (Walczak *et al*, 1999; Kim *et al*, 2001b), this death ligand could be used in combination with IR in anticancer treatment. Therefore, we analysed whether the combined actions of TRAIL and IR effectively overcome the clinically known resistance of human RCCs against induction of apoptosis.

The data presented here demonstrate expression of apoptosis-inducing TRAIL-R1- and -R2-receptors in all RCC cell lines. Moreover, recombinant TRAIL was capable of inducing marked apoptosis in three out of eight cell lines. This observation was important in that it proved the existence of a functionally active mechanism of self-destruction in a significant proportion of RCCs, provided that appropriate stimuli can be applied or sensitivity to weak stimuli can be raised. Despite the presence of proapoptotic TRAIL-R1 and -R2 receptors in all cell lines, however, therapeutically relevant doses of IR combined with TRAIL treatment largely failed to increase the apoptotic response in most of our RCC cell lines. Thus, a sensitisation effect with marked enhancement of apoptosis after combined TRAIL and IR treatment was observed in one out of six RCC cell lines only. These observations were at variance with much more favourable recent reports on synergistic effects of TRAIL and IR in breast and colon carcinoma cell lines (Chinnaiyan *et al*, 2000; Ravi *et al*, 2001) as well as Jurkat cells (Gong and Almasan, 2000; Kim *et al*, 2001a). It has to be considered, however, that only short-term effects of TRAIL and IR becoming evident within 72 h could be assessed under the conditions of our experiments, whereas potential long-term effects of IR were not within the scope of our investigation.

Little is known concerning the mechanisms underlying the resistance to TRAIL and/or IR. However, it is thought that apoptosis induction in response to IR requires an intact p53 system (Lowe *et al*, 1993), and, accordingly the synergistic effect of TRAIL and IR in breast carcinomas was reported to depend on a p53 wild-type status (Chinnaiyan *et al*, 2000). One possible mechanism promoting the synergistic effect of TRAIL and IR might involve the upregulation of TRAIL-R2, known to be a downstream effector gene of p53 (Yu *et al*, 1999). Actually, irradation resulted in an increase of TRAIL-R2 expression in clearCa-22 (which does not harbour mutations in p53-coding regions), thereby possibly rendering this cell line more sensitive to TRAIL-induced apoptosis. Nevertheless, the overall level of apoptosis induction in clearCa-22 after combined exposure to TRAIL and IR was rather low (maximal decrease of cell number: 46.8±1.5% of the control) when compared with the effects observed in a colon carcinoma cell line (Ravi *et al*, 2001) and in different clones of Jurkat cells (Belka *et al*, 2001; Kim *et al*, 2001a).

According to current concepts, apoptosis can be induced via at least two distinct, but interconnected pathways that activate different initiator caspases, that is, caspase-8 and -9, which converge at the level of executioner caspase-3, -6, and -7. The simultaneous activation of *both* pathways is thought to result in a strong amplification of the original apoptosis signal (Li *et al*, 1998; Belka *et al*, 2000,^2001^; Suliman *et al*, 2001; Joseph *et al*, 2002). We, therefore, analysed the activation status of these two principal pathways in clearCa-22 after exposure to TRAIL or IR alone and in combination. In these investigations, we could demonstrate cleavage of the executioner caspase-3 in clearCa-22 only after combined exposure to TRAIL and IR, whereas exposure to TRAIL or IR alone resulted in no or barely detectable caspase-3 cleavage only. In accordance, cleavage of PARP, known to be a substrate of active caspase-3 (Yang *et al*, 2001), became evident in clearCa-22 only after combined exposure to TRAIL and IR. Therefore, activation of caspase-3, which was previously shown to be involved in TRAIL- and IR-induced apoptosis in other tumour types (Griffith *et al*, 1998; Gong and Almasan, 2000; Yamanaka *et al*, 2000; Belka *et al*, 2001; Joseph *et al*, 2001, 2002; Kim *et al*, 2001a; Nimmanapalli *et al*, 2001; Rudner *et al*, 2001; Suliman *et al*, 2001; Sun *et al*, 2001; Joseph *et al*, 2002), is also found in RCCs. At variance with other tumour types (Coelho *et al*, 2000; Sun *et al*, 2001; Yang *et al*, 2001), however, no cleavage of executioner caspase-6 or -7 became evident in our RCC model system.

Since caspase-3 can be activated by both principal apoptosis signalling pathways involving the initiator caspase-8 or -9, respectively, we next analysed the functional contribution of these distinct apoptosis pathways. Thus, we could demonstrate a strong cleavage of the initiator caspase-8 in clearCa-22 after exposure to TRAIL or IR alone and in combination. These findings are in line with reports demonstrating caspase-8 cleavage after exposure to TRAIL or IR in RCCs (Pawlowski *et al*, 2000) and many other tumour types, including carcinomas of the liver, prostate and lungs (Yamanaka *et al*, 2000; Nimmanapalli *et al*, 2001; Sun *et al*, 2001), melanoma (Griffith *et al*, 1998) and Jurkat cells (Gong and Almasan, 2000; Belka *et al*, 2001; Kim *et al*, 2001a; Rudner *et al*, 2001; Suliman *et al*, 2001). Activation of caspase-8 by TRAIL or IR, therefore, seems to be a common reaction pattern in a variety of different tumour types, including RCCs. Of note, a marked downregulation of c-FLIP protein, which is able to inhibit caspase-8 activation (Irmler *et al*, 1997), became evident in clearCa-22 after exposure to TRAIL and/or IR. Although the role of c-FLIP in inhibiting TRAIL-induced apoptosis is controversial (Griffith *et al*, 1998; Pawlowski *et al*, 2000; Wang *et al*, 2000; Mitsiades *et al*, 2001), these results suggest that reduced c-FLIP might be involved in the activation of caspase-8 in RCCs.

Activation of initiator caspase-9 after exposure to TRAIL or IR was reported in carcinomas of the prostate and lung (Nimmanapalli *et al*, 2001; Joseph *et al*, 2001; Sun *et al*, 2001), Ewing's sarcoma (Mitsiades *et al*, 2001) and Jurkat lymphoma cells (Kim *et al*, 2001a; Rudner *et al*, 2001). In contrast, we could not detect any cleavage of inactive procaspase-9 into active caspase-9 in clearCa-22 after exposure to TRAIL and/or IR. Therefore, transduction of the apoptosis signal via the ‘mitochondrial’ caspase-9 pathway seems to be blocked in our RCC model system. Our investigation suggests that lack of Bid cleavage might contribute to the failure of caspase-9 activation. Thus, it is known that active caspase-8 is able to cleave Bid into tBid, which translocates to mitochondria, causing cytochrome *c* release and activation of caspase-9 as well as caspase-3 (Li *et al*, 1998; Belka *et al*, 2001; Kim *et al*, 2001a; Nimmanapalli *et al*, 2001; Suliman *et al*, 2001). This interconnection between the caspase-8 and -9 pathway via Bid is believed to represent a strong amplification loop of the apoptosis signal. However, no cleavage of Bid was detectable in clearCa-22 after exposure to TRAIL and/or IR despite cleavage of caspase-8 and -3. Another recently identified regulator of caspase-9 and Bid is Akt, a serine/threonine protein kinase, which is activated by phosphorylation. Interestingly, the expression of active Akt was found to correlate with TRAIL resistance in prostate carcinomas (Thakkar *et al*, 2001). Akt exerts its antiapoptotic effects through caspase-9 inactivation by phosphorylation as well as direct inhibition of Bid cleavage (Thakkar *et al*, 2001). In clearCa-22, however, expression of active Akt was present neither before nor after exposure to TRAIL and/or IR. In contrast to other tumour types, therefore, Akt seems to be of minor importance for inhibiting Bid cleavage and preventing caspase-9 activation in our RCC model system.

Moreover, a high basal expression level of the IAP family member XIAP became evident in our RCC cell line clearCa-22. Importantly in this context, we observed the appearance of a cleavage product of XIAP after exposure to TRAIL and/or IR. Since the fragment of XIAP contains the BIR3-Ring domain, which was shown to inhibit specificially the activation of caspase-9 (Deveraux *et al*, 1999), this cleavage product might also be involved in the deficient activation of caspase-9 in our cell line. In contrast, expression of survivin, another member of the IAP family, which was reported to be downregulated in prostate carcinoma cells after exposure to TRAIL (Nimmanapalli *et al*, 2001), was not affected in clearCa-22 after exposure to TRAIL and/or IR.

Collectively, our results demonstrate a marked heterogeneity in the responsiveness of human RCC cell lines to TRAIL-mediated apoptosis. Moreover, IR resulted in a sensitisation to TRAIL-induced apoptosis in one RCC cell line only. In addition, our observations suggest that TRAIL- and IR-induced apoptosis in RCC is predominantly mediated via the caspase-8 pathway, whereas the caspase-9 pathway is not utilised. The missing activation of the caspase-9 pathway may result from an impaired ‘cross talk’ with the caspase-8 pathway because of the lack of Bid cleavage and from the appearance of as XIAP cleavage product known to inhibit caspase-9 activation. Therefore, the deficient activation of caspase-9 might contribute to the clinically known resistance of human RCC against IR and also argues against an effective combination therapy with TRAIL and IR in this tumour type.
